# Open surgical management of a high-flow supratentorial pediatric pial arteriovenous fistula

**DOI:** 10.3171/2025.7.FOCVID25107

**Published:** 2025-10-01

**Authors:** Lucinda Chiu, Jillian Plonsker, Ali Shaibani, Jonathan Scoville, Sandi Lam

**Affiliations:** 1 Department of Neurosurgery, Division of Pediatric Neurosurgery, Ann & Robert H. Lurie Children’s Hospital of Chicago, Northwestern University Feinberg School of Medicine, Chicago;; 2 Department of Neurological Surgery, Rush University Medical Center, Chicago; and; 3 Department of Radiology, Ann & Robert H. Lurie Children’s Hospital of Chicago, Northwestern University Feinberg School of Medicine, Chicago, Illinois

**Keywords:** color Doppler, indocyanine green, pial arteriovenous fistula, pediatric, venous varix

## Abstract

Pial arteriovenous fistulas (pAVFs) in pediatric patients are a rare, usually congenital, high-flow vascular anomaly. Because of their rarity, there are no consensus treatment guidelines. Although most pAVFs can be treated endovascularly, surgical treatment has yielded higher obliteration rates. Superficial pAVF varix location and angioarchitecture difficult for endovascular flow occlusion can also make resection more favorable. Here the authors present the case of an incidentally diagnosed left posterior parietal pAVF in a 14-year-old boy, which was treated successfully with a craniotomy and fistula ligation.

The video can be found here: https://stream.cadmore.media/r10.3171/2025.7.FOCVID25107

## Transcript

This video presents the open surgical management of a high-flow supratentorial pial arteriovenous fistula in a pediatric patient.

### 0:30 Clinical Presentation and Neurological Examination.

A 14-year-old boy presented with 4 days of persistent, intermittent frontal headaches after minor head trauma. The patient has a past medical history of hereditary hemorrhagic telangiectasia. On presentation, the patient is neurologically intact.

### 0:46 Neuroimaging and Choice of Approach.

Digital subtraction angiography confirms this vascular lesion is most consistent with a pAVF, with a hypertrophied arterial feeder from the superior temporal branch of the left middle cerebral artery, which enters at the caudal aspect of the 2.3 × 4.4 × 4.2–cm varix, and venous drainage through a singular superficial cortical vein to the superior sagittal sinus.^1^

Given the presence of a radiographically confirmed high-flow vascular lesion with a bulky venous varix deforming the overlying skull and underlying brain parenchyma with surrounding T2 signal changes on MRI, open surgical intervention was favored following multidisciplinary neurovascular conference and discussion with the patient and his parents.

Multiple approaches were considered.^2^ While many pAVF cases are amenable to endovascular management, endovascular embolization was not chosen given the high-flow lesion, large superficial varix, and the likely bulky coil/embolysate mass that may contribute to a compressive lesion in a sensorimotor anatomical location.^3^

Radiosurgery was not pursued given the large size of the venous varix with underlying mass effect on brain parenchyma, as well as the sizable arterial feeder.^4^ Medical management and/or observation is not recommended given the high lifetime risk of rupture without definitive treatment.^5^ Open surgical treatment was ultimately chosen for this young, healthy patient, as it allows for definitive removal of this surgically accessible, superficially located vascular lesion to decrease hemorrhage risk and optimize neurocognitive development caused by varix compression.^5^

### 2:22 Positioning and Surgical Equipment.

The patient was positioned in a right lateral decubitus position. The head was fixed in a Mayfield skull clamp.

We used the option of neuromonitoring for the sizable high-flow lesion posterior to the sensorimotor area. Stereotactic neuronavigation was used to plan the craniotomy. A microscope, intraoperative ultrasound, and indocyanine green were used to assist with identifying the vascular anatomy and resecting the lesion.

Anesthetic considerations included arterial line insertion and keeping the systolic blood pressure between 100–120. Given the risk of bleeding during high-flow open pAVF surgical resection, a risk which is further increased in the pediatric population due to their lower circulating blood volume, appropriate preoperative labs should be obtained and blood should be available in the operating room prior to the surgery.

### 3:13 Surgical Procedure.

A left posterior parietal craniotomy was performed.

Preoperative CT head shows scalloping of the undersurface of the skull from the pAVF venous varix (as shown by the solid arrow) and the dilated draining vein (as shown by the dashed arrow). Intraoperatively, this was seen as an indentation on the undersurface of the bone flap. Pial AVFs in pediatric patients more commonly present with a venous varix, which can lead to calvarial remodeling due to their congenital, long-standing nature.^4,6^ Thus, it is critical to map out the varix with relation to the planned craniotomy, and to take care when drilling due to these bony irregularities. Violation of the high-flow venous varix during the craniotomy can quickly lead to exsanguination of the patient, particularly in pediatric patients who have a lower circulating total blood volume.

Baseline SSEP/MEP were obtained and monitored throughout the case. Dura was opened carefully to keep the vascular lesion safe, and the pAVF was visualized.

A baseline ICG run was performed, and shows the arterial feeder, venous varix, and draining vein going to the sagittal sinus. Intraoperative color Doppler was used as an adjunct to confirm the exact fistulous point.

The arterial feeder is dissected out and a temporary aneurysm clip is placed on the arterial feeder. The pAVF is skeletonized, working caudally to cut off the arterial feeders. The arterial feeder is cauterized and divided. Further pAVF dissection is performed. The venous varix appears more deflated and dark in color. The draining vein, which appears smaller and collapsed, is then dissected out.

### 5:03 Resection of the Lesion.

The draining vein was cauterized and divided. The venous varix is further dissected, and the pAVF is removed. SSEP and MEP remained at baseline at time of closing.

### 5:16 Postoperative Neuroimaging and Postoperative Clinical Course.

The patient tolerated the procedure well.

Postoperative angiography on postop day 1 showed complete pAVF obliteration. The patient was discharged home on postop day 2. At 1-year follow-up, the patient remains neurologically intact, has returned to baseline at school, and has resumed all physical activities. His MRI/MRA brain shows no residual or recurrence of his pAVF.

## Acknowledgments

We thank Jesse Arseneau for his assistance with the editing of this surgical video.

## Disclosures

Dr. Lam reported personal fees from Brainlab, Jaguar Therapeutics, Ovid Therapeutics, Encoded Therapeutics, Livanova, and Synergia outside the submitted work.

## Author Contributions

Primary surgeon: Lam. Assistant surgeon: Scoville. Editing and drafting the video and abstract: Lam, Chiu, Scoville. Critically revising the work: Lam, Chiu, Plonsker, Scoville. Reviewed submitted version of the work: all authors. Approved the final version of the work on behalf of all authors: Lam. Supervision: Lam, Plonsker.

## Supplemental Information

### Patient Informed Consent

The necessary patient informed consent was obtained in this study.
